# Current scenario and future perspectives of clinical research in Brazil: a national survey

**DOI:** 10.3332/ecancer.2023.1640

**Published:** 2023-11-23

**Authors:** Heloisa Resende, Taiane F Rebelatto, Gustavo Werutsky, Gustavo Gossling, Vinícius Q Aguiar, Guilherme M C Lopes, Biazi R de Assis, Lilian Arruda, Carlos H Barrios

**Affiliations:** 1 Associação Instituto Projeto Cura, São Paulo 05507-020, Brazil; 2 Latin American Cooperative Oncology Group, Porto Alegre 90619-900, Brazil; 3 Centro Universitário de Volta Redonda, UniFOA, Volta Redonda 27240-560, Brazil; 4 Hospital Hinja, Volta Redonda 27251-260, Brazil; 5 Hospital São Camilo, São Paulo 17580-000, Brazil; 6 Grupo Oncoclínicas, São Paulo 04543-906, Brazil; a https://orcid.org/0000-0003-4692-3743; b https://orcid.org/0000-0001-7306-5428; c https://orcid.org/0000-0001-6271-105X; d https://orcid.org/0000-0002-4361-2889; e https://orcid.org/0000-0002-6257-0119; f https://orcid.org/0000-0002-5654-3579; g https://orcid.org/0000-0002-2727-5472; h https://orcid.org/0000-0002-7101-4325; i https://orcid.org/0000-0001-6021-667X

**Keywords:** cancer, survey, clinical research, clinical trials, barriers

## Abstract

**Background:**

Epidemiological and clinical cancer research is essential to understanding tumour behaviour and developing new therapies in oncology. However, several countries including Brazil as well as many other regions of the world have limited participation in cancer research. Despite 625,000 new cancer cases recorded in Brazil in 2022, only 2.2% of ongoing cancer clinical trials are available in the country. We conducted an online survey to describe physician engagement with research and to identify the main barriers precluding participation in and conduct of clinical cancer research in the country.

**Methods:**

An anonymous online survey of 23 objective questions was sent by e-mail to Brazilian members of the Latin American Cooperative Oncology Group and the Brazilian Society of Clinical Oncology. The first 13 questions addressed demographic information, medical training and previous research participation. In the second part, the main barriers to engagement and participation in clinical trials in Brazil were addressed. Continuous variables were measured by median and range. Analyses were performed using SAS statistical software (version 9.4; SAS Institute, Inc. Cary, NC).

**Results:**

109 physicians answered the survey. Most participants were oncologists (*N* = 98, 89.9%), living in capital cities (*N* = 84, 77.1%), were from the Southeast region of Brazil (*N* = 63, 57.8%) and worked at institutions providing exclusively private healthcare (*N* = 59, 54.1%). Of the 109 respondents, 83 (76.1%) reported working in research centres (as investigators or sub-investigators). Surprisingly, 31.2% of physicians recognised they invite less than 1% of their patients to participate in clinical trials, even though 98 (89.9%) considered the participation of patients in clinical trials extremely relevant. The main barriers compromising the conduct of research in the country were the low number of available trials (48.2%) and the lack of qualified human resources to staff research sites (22.9%). Other reported barriers were the lengthy regulatory approval process (42.2%), followed by a lack of awareness of clinical research by patients resulting in low recruitment rates (24.1%). Of the 26 (23.8%) respondents not working with research, 25 (96.1%) reported interest in being involved, 31.8% have tried participating in research and 62.4% reported limited knowledge of trial procedures.

**Conclusion:**

These results suggest a clear need to further engage physicians in clinical research activities in Brazil. Patient education strategies should improve the low recruitment rates and secondarily increase the number of proposed trials in the country.

## Background

Cancer is the first or second major cause of death in 112 of 183 countries, accounting for 18.1 million new cases and 10 million deaths annually worldwide [1, 2]. In Brazil, cancer is the second cause of death, with 625,000 new cases and 225,830 deaths expected annually [3]. Advances in cancer control involving prevention, early diagnosis and treatment strategies have improved the prognosis of the disease. These advances were made possible by heavy investments in epidemiological and clinical research.

Epidemiological research aims to understand a tumour's clinical, molecular and pathological features and may identify gaps in cancer care in each region. This information is essential for policy and decision-making and determining which tumour molecular alteration should be prioritised for diagnostic tests and drug development in clinical research.

Clinical trials are essential to developing new oncological therapies [4]. In the last decades, it has become evident that clinical trials should include a wider participation of patients from different regions of the world and with different ethnic backgrounds to guarantee the representability of all populations, reduce costs and accelerate recruitment [5]. Notably, from 10,098 ongoing oncology clinical trials in 2022, less than 10% are available in Latin American (LATAM) countries [6]. Another analysis showed that 87% of all ongoing clinical trials are conducted in the United States (USA), Canada, Europe and Australia. Consistently, a very low percentage of trials are available in other countries [7], reflecting the low representation of populations from many world regions in ongoing oncology clinical trials. The increase in participation of underrepresented populations in clinical trials is essential to further advance therapeutic results in oncology.

A recent report showed that Brazil ranks 19th in oncological clinical trial participation [8]. Brazil has attractive characteristics for oncological research, such as a high number of patients with cancer, 75% covered by the Public Health System (SUS) [9]. The population has broad ethnic diversity comprising descendants from indigenous, African, European and Asian populations [10]. Additionally, the country has significantly improved in terms of technological and human resource research infrastructure in the last decade. However, there are challenges to be overcome to increase Brazilian participation in cancer clinical research. Regulatory approvals are conducted by national regulatory agency Agência Nacional de Vigilância Sanitária (ANVISA) and the federal research committee Comissão Nacional de Ética em Pesquisa (CONEP). ANVISA has developed a reliable work and has conducted its actions within strategic planning, which is reformulated each 4 years, and aims align Brazilian regulatory processes with best international practices to bring predictability and shortening regulatory times [11, 12]. CONEP, since its creation in 1996, also has adopted conceptual and consistent ethical grounds of regulations [13]. Even though both agencies have worked to improve regulatory environment in Brazil, bottlenecks remain, as the long time for regulatory processes. Other crucial point is scarcity of governmental funding in cancer research. The global Brazilian health expenditure in 2019 was 9.6% of its gross domestic product, however only 3.9% is provided by government, with 5.7% provided by private sector [14]. This small expenditure by the public sector in health, results in few or no investment in cancer clinical research featuring a landscape with isolated sources of financing as Programa Nacional de Apoio a Atenção Oncológica created in 2014, which represents one opportunity for cooperative groups to conduct academic projects [15, 16]. However, it is clearly insufficient to guarantee that local and relevant questions are encompassed [17], and not even give wide access to patients participate in.

To understand the current level of engagement of Brazilian oncologists with clinical research in Brazil and also to describe the barriers faced by physicians to participate in clinical trials, the CURA Institute, in partnership with Latin American Cooperative Oncology Group (LACOG), proposed a survey to Brazilian clinical oncologists. The CURA Institute, based in Brazil, is an organisation in Latin America dedicated to planning and carrying out awareness campaigns and fundraising drives to support research into combating cancer. LACOG is a non-profit organisation, exclusively dedicated to epidemiological, clinical and translational cancer research in LATAM.

## Methods

An online survey of 23 objective questions was developed (Supplementary material). The objective was to describe the level of engagement of Brazilian oncologists and describe barriers faced by physicians to participate in cancer clinical trials. The first 13 questions addressed physician’s demographic information, medical training, previous clinical research experience and patient participation in clinical research. In the second part of the survey, physicians were divided into two groups: 1) physicians who work as investigators in a research site, with five additional questions to identify barriers to participation in and performance of clinical research; 2) physicians who do not work with clinical trials, with five questions addressing the causes and barriers preventing their participation in clinical trials.

The survey link was sent by e-mail to 350 Brazilian members of the LACOG and 2,300 members of the Brazilian Society of Clinical Oncology (SBOC) from May 04, 2022, to June 05, 2022. The study population consisted of physicians who work in Brazil with cancer and belong to LACOG or SBOC. The questionnaire was anonymised according to current legislation. The estimated time required to answer the questionnaire was 10 minutes. A brief explanation of the survey proposal containing objectives, population and ethics information was displayed in the first part. No financial incentive was offered to responders. The complete questionnaire is presented in the Supplementary material section. The IRB of Fundação Osvaldo Aranha (UNIFOA, Rio de Janeiro/Brazil) approved this study (Approval protocol number: 53646121.1.00005237).

### Statistical analysis

Answers to the 23 questions were summarised by absolute and relative frequencies. Continuous variables were measured by median and range; categorical variables were treated as proportions. Chi-Squared tests were used to investigate the association between variables. A logistic regression was applied to analyse which variables were associated with recruiting patients to clinical trials and working as investigators. All analyses were performed using SAS statistical software (version 9.4; SAS Institute, Inc. Cary, NC). A significance level of 5% was considered.

## Results

A total of 109 physicians who lived and worked in Brazil responded to the survey. The median age was 42 years (range 30–76). Most participants were male (*N* = 61, 56%), oncologists (*N* = 98, 89.9%), had completed specialty training more than 10 years ago (*N* = 61, 56%), lived in capital cities (*N* = 84,77%), and were from the Southeast region of Brazil (*N* = 63, 57.8%). Participant characteristics are described in Table 1.

Most respondents worked in institutions providing exclusively private healthcare (*N* = 59, 54.1%), and 35 (32.1%) in institutions providing private and public healthcare. A total of 92 (84.4%) physicians reported that their institution had an associated research centre, with 90 (97.8%) of them conducting clinical trials. Even though 89.9% of respondents considered the participation of patients in clinical trials extremely relevant, 31.2% of physicians invite less than 1% of their patients to participate in clinical trials and 47.7% reported that less than 1% of patients are included in trials (Table 1). No previous experience with clinical research during the specialty training was reported by 32.1% (*N* = 35) of physicians surveyed.

**Figure 1.**
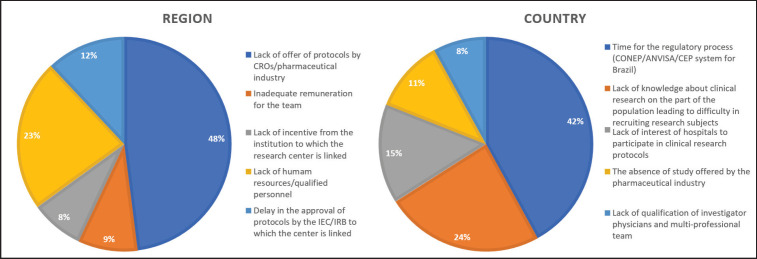
Main barriers to conducting clinical research in the region and in the country.

Of 109 physicians, 83 (76.1%) reported working in a research centre as investigators or sub-investigators. Regarding the main barrier to conduct clinical research, 48.2% referred a low number of available clinical trials, followed by 22.9% who reported a lack of qualified human resources (Figure 1). Regarding the main barrier in Brazil, the lengthy approval period by regulatory organs was reported by 42.2% of respondents. Importantly, a lack of clinical research awareness by the population resulting in low recruitment levels was noted by 24.1% (Figure 1).

From the respondents' point of view, clinical research is still not well developed in Brazil, it is still concentrated in capital cities, and with few available clinical trials.

**Figure 2.**
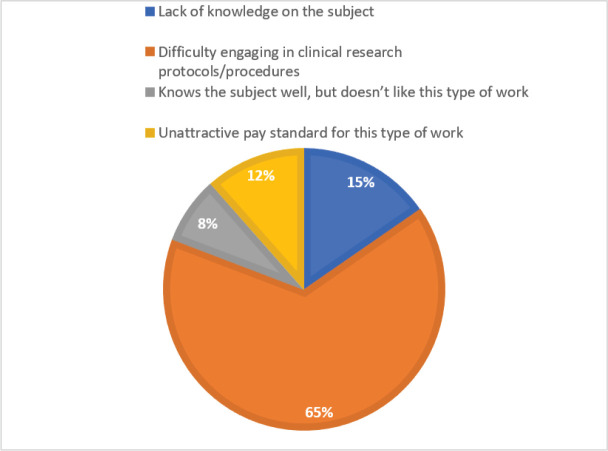
Reasons for not working in clinical research.

Of 26 (23.8%) participants who do not work in research centres, 96% reported interest in working with research. However, only 31.8% have tried participating in research, and 65.3% reported limited protocol and trial procedures training (Figure 2). Regarding interest in clinical research of the hospitals where they worked, 21 (80.8%) of the oncologists answered that hospitals are interested in participating in clinical research and have few human resources, but there are barriers that hamper their participation.

Having contact with clinical research during training was associated with working in a capital (*p* 0.0167), age less than 50 years (*p* 0.0059), and finishing oncological training in the last 5 years (*p* 0.0142), only in the univariate analysis model. Regarding working in research centres, none of the analysed factors (age, gender, time since specialisation conclusion, had contact with clinical research during medical degree, health service and living in a capital) were associated with working at a research centre (Table 2). Referring more than 5% of patients to participate in clinical research, was associated with working as a principal investigator or sub-investigator (RR 0.43, *p* = 0.05) and being linked to a research centre (RR 0.27 *p* = 0.02) only in the univariate analysis model. In the multivariate analysis, referral of more than 5% of patients was not associated with age, gender, time since specialisation conclusion, had contact with clinical research during a medical degree, health service, living in a capital, working as a principal investigator or sub-investigator and being linked to a research centre (Table 3).

## Discussion

The present study aimed to understand the current level of engagement of Brazilian oncologists with cancer clinical research, describing the barriers to participation in clinical trials. We also investigated factors associated with working in research centres and the referral of patients for clinical trials.

The survey link was sent simultaneously to members of a research organization (LACOG) who lived and worked in Brazil and a professional organization (SBOC), which are representative entities in the Brazilian context. The lower response rate among members might reflect the lack of interest or the barrier to engaging in clinical trials in Brazil. Among all respondents, 57% worked in the Southeast region and 77% in capitals, possibly reflecting a concentration of specialists in these settings in Brazil.

Contact with clinical research was described during graduate training, as reported by 68% of interviewees. The rate of oncologists aged 50 years or older with contact with clinical research during graduate training was lower than those younger than 50. In line with that, physicians who finished their training in the last 5 years had more contact with clinical research than those who completed their training more than 10 years ago. This finding may reflect the recent inclusion of research as a subject for oncology trainees. However, having contact with research during the graduate training was not associated with offering participation in clinical research to patients. Most physicians (61.4%) offer participation in clinical trials to less than 5% of patients, and one third of them offer participation to less than 1%. Regarding effective participation of patients, half of oncologists reported that less than 1% of their patients participate in clinical research. This may suggest that there is low interest in referring patients to clinical research and at the same time that there is a low number of protocols available. This evidence also supports the view that there is a very low number of Brazilian patients participating in clinical trials. It should be noted that in the USA, despite the high number of new cancer cases annually and high offer of clinical trials in the Country, only 3%–5% of cancer patients participate in clinical research [18].

Some common aspects in Brazil and USA are patients' lack of awareness of clear information about clinical trials participation and distrust in the medical system [19]. In Brazil, additional barriers make the referral of patients to clinical trials more challenging than in the USA and other high-income countries. Physicians and patients have difficulties finding updated information on ongoing clinical trials. The Plataforma Brasil allows finding research through search title, principal investigator, proponent institution, initial and final dates and contact [20]. However, it is not possible to search for specific trials. Some initiatives made by SBOC and private platforms try to facilitate the integration between physicians and available clinical trials. The SBOC platform is available on the society website and access is restricted to members. It comprises protocol title, pathology, research centre and state [21]. However, it is available only in the restricted area to associates [6, 21]. The development of a broadly available platforms to inform about all available clinical trials will be a major advance and would facilitate the referral of patients. Besides that, the platform should contemplate information to patients, raising public awareness about clinical research.

Most interviewed physicians (54.1%) actively working in clinical trials are in the private system, although in Brazil, 75% of the population is covered only by public healthcare [9]. Initiatives adopted by Oncoclínicas Group (GOC), a private oncology group, are attempting to improve the number of research units in the country. GOC has implemented a research program, raising the number of research centres in Brazil, in many regions of the Country. The strategy is focused on training and maintaining qualified human resources, creating a central office to concentrate processes and adopting common standard procedures [22].

Confirming the impression of concentration of clinical trials in a few cities, 77% of the respondents that work in clinical research reported living in capital cities. In the Brazilian public system (SUS), cancer care is offered in reference hospitals with government certification [23, 24]. In the national territory, 317 units/centres are localised in reference hospitals of each region, able to offer cancer care. Of note, 58% of these units/centres are localised outside capital cities, which compromises access to clinical trials [25]. One of the aspects of a sustainable clinical research program is to offer clinical trials participation to patients at the local level [18].

The survey didn't investigate why physicians do not offer clinical trial participation for their patients. However, when we analysed the characteristics of respondents, a significant percentage of referrals were by physicians working in a hospital with clinical research sites and oncologists working as principal investigator or sub-investigator (univariate analysis-Table 3). This reinforces aspects commented by other authors that logistical barriers, such as transport, discourage referring patients to clinical trials [26]. Other reasons also considered are bias of recruitment when the physician considers the case too complex and decides not to refer the patient. Competing patient care demands in public hospitals with scarce resources are also important to consider [27, 28].

Is clear that physicians are one of the main aspects to target. Therefore, it is necessary to increase their awareness of the benefits of clinical research programs and engage them as active recruiters and players that promote clinical research participation [18].

Oncologists reported that they do not work with clinical research (one-quarter of the respondents). However, almost 100% of them answered that they are interested in participating in clinical research activities. However, only 20% referred to having tried to include their institution in clinical research, mostly unsuccessfully. This perception also has been pointed out by Piccart *et al* [4] when they explain that in the ideal world, all interested cooperative groups should be able to participate in a clinical trial. However, it rarely happens in the real world. The high cost of clinical research in oncology and complexity of approval process are many times drivers to decision about which countries and groups will participate in clinical trials. Therefore, pharmaceutical companies will choose the countries and groups based on previous experience, logistical aspects and budget constraints, living new centres without the opportunity to participate. LACOG has developed a great work regarding fostering and improving access to cancer research, which utmost can result in increasing of rate patients’ referral by physicians and increasing of physicians engaged in cancer clinical research. LACOG is a multicentre collaborative cancer group founded in 2008, with most of members from Brazil, but there are members from other LATAM countries, its coordinating office is located in Porto Alegre, Brazil [29]. LACOG has presented expressive growing in the last years, participating in 31 studies and completing 330 site activations by the last year, also representing a cooperative group in AL with a major number of scientific publications. LACOG has assisted investigators with their study concept, protocol development and management, monitoring, data management, pharmacovigilance and statistical analysis. Also, LACOG has developed its own research projects which are sponsored by pharmaceutical companies and governmental grants. Currently, LACOG manages tumour groups (breast, digital, gastrointestinal, genitourinary, genitourinary, geriatric, gynaecological, head and neck, lung, neuro, radiation and sarcoma) [30]. As a cooperative group LACOG has been recognised as a renowned institution providing support and networking for investigators.

Half of respondents in both groups (working and not working in clinical research) considered clinical research underdeveloped in Brazil, restricted to capital cities and conducted mainly by opinion leaders. Although Brazil is the country in South America with higher participation in clinical trials, followed by Argentina and Chile [31] its participation is only 2.2% of the ongoing clinical trials worldwide [8]. Concerning South America participation in cancer clinical research, the most of clinical trials available are phase III (around 70%) and pharma industry sponsored trials [31, 32]. Participation in early phase II clinical trials is around 20% and phase I is less than 2% [31]. Although the participation is increasing in the last years, it is predominantly in trials of confirmatory approval drugs.

Oncologists actively working on clinical research, pointed out low availability of clinical trials and lack of qualified human resources as two main barriers that hamper major participation in the region. Regarding the national scenario, a long approval period by regulatory agencies, was also pointed out by most of them. Of note, the duration of the regulatory process in Brazil involving the ANVISA, local institutional review boards Comitês de Ética em Pesquisa (CEPs), and CONEP, has had a mean period of approval of 215 days on average which is longer than the approval time in Argentina (113 days) and much longer than in the USA (32 days) [8]. One of the aspects that might explain the lengthy approval process in Brazil is the necessity of double approval by review boards, local and federal, (CEP and CONEP). The approval process is an increasingly critical point in Brazil. Although these timelines have been shortened, it is necessary continuum commitment to become them more competitive [33, 34]. The significant current complexity of trials, especially related to precision medicine involving biological samples requires a more complex and consequently more prolongated and costly approval process. The inclusion of nontraditional markets, such as Asia and Latin America, to guarantee fast and adequate recruitment is an important aspect in the current global research strategy. Due to these needs, investments in lower-middle income countries have increased 4%–16% in the 2005 to 2012 period [5], and Brazil has not seen this growth, having a stable and low participation in clinical trials. The second cause, uniformly pointed out, was the population's lack of clinical research awareness, resulting in low recruitment levels noted by one quarter of respondents. This result certainly identifies the lack of the population awareness regarding clinical research benefits and its importance.

The present survey has limitations that need to be addressed. The low response rate obtained from LACOG and SBOC members might reflect the limited access to clinicians to information regarding research. Other limitations are memory bias linked to survey methodology and inability to report reasons for the low referral of patients to clinical trials.

However, to our knowledge, this is the first survey addressing physician’s perspectives on clinical cancer research in the country. It clearly identifies two main barriers that may explain the low Brazilian participation in clinical trials. Information and educational factors including patients and physicians [35, 36], and government-related barriers, reflect the long and bureaucratic regulatory approval processes.

## Conclusion

The survey points to very specific aspects that need to be addressed by medical societies and non-government organisations to modify the scenario. Thus, actions to promote information, training and education of both physicians and patients are desirable. In parallel, efforts looking to improve and qualify legislation and government processes are also mandatory to promote the country in the competitive world of clinical research. In that regard, new legislation is being proposed and is currently under discussion to facilitate and speed regulatory approvals. This can have a significant impact on the number of trials that would become available for Brazilian patients. At the same time, participation in early clinical trials (phase I and II), which would become available with shorter approval times, would be an important step forward in generating experience and furthering the qualification of investigators and research sites.

## Conflicts of interest and funding

Heloisa Resende has received research funding from Novartis and Roche, all outside the scope of this manuscript.

Taiane Francieli Rebelatto has no relationships to disclose.

Gustavo Werutsky has received research funding from AstraZeneca/MedImmune, Bristol-Myers Squibb Brazil, GlaxoSmithKline, Lilly, Novartis, Pfizer, Roche and Roche/Genentech, all outside the scope of this manuscript. Has consulting or advisory role at Merck.

Gustavo Gössling has received research funding from AstraZeneca/Merck and Janssen Oncology, all outside the scope of this manuscript.

Vinícius Aguiar has no relationships to disclose.

Guilherme Lopes has no relationships to disclose.

Biazi Assis has no relationships to disclose.

Lilian Arruda has no relationships to disclose.

Carlos H Barrios has received research funding from AB Science, Abbvie, Abraxis BioScience, Amgen, Asana Biosciences, Astellas Pharma, AstraZeneca, Biomarin, Boehringer Ingelheim, Bristol-Myers Squibb, Celgene, Clinica Atlantis, Covance, Daiichi Sankyo, Exelixis, GlaxoSmithKline, Halozyme, ImClone Systems, INC Research, inVentiv Health, Janssen, LEO Pharma, Lilly, Medivation, Merck, Merck KGaA, Merrimack, Millennium, Mylan, Novartis, Pfizer, PharmaMar, Polyphor, Roche/Genentech, Sanofi, Shanghai Henlius Biotech and Taiho Pharmaceutical, all outside the scope of this manuscript. Has consulting or advisory role at AstraZeneca, Boehringer Ingelheim, Eisai, GlaxoSmithKline, Libbs, Lilly, MSD Oncology, Novartis, Pfizer, Roche/Genentech and United Medical. Has stock and other ownership interests in MedSIR and Tummi.

## Funding

The authors would like to thank Hoffmann-La Roche Ltd, Switzerland for supporting this research.

## Figures and Tables

**Table 1.** Participant characteristics.

Characteristics	*N* (%)
Age – Median (Min–Max)	42 (30–76)
30–40 years	45 (41.3)
40–50 years	48 (44.0)
≥50 years	16 (14.7)
Gender	
Female	48 (44.4)
Male	61 (56.0)
Specialty	
Head and neck surgery	1 (0.9)
Oncological surgery	1 (0.9)
Mastology	3 (2.7)
Oncology	98 (89.9)
Pathology	2 (1.8)
Radiotherapy	4 (3.7)
Time since specialisation conclusion – Median (Min – Max)	12 (1–42)
≤5 years	18 (16.5)
5–10 years	30 (27.5)
>10 years	61 (56.0)
Region	
Southeast	63 (57.8)
South	12 (11.0)
Central-West	10 (9.2)
North	3 (2.7)
North-east	21 (19.3)
Live in capital city	
Yes	84 (77.0)
No	25 (23.0)
How relevant do you consider the participation of your patients in clinical research for the quality of care in oncology?	
Very relevant, as the involvement of the patient in clinical research will help the doctor and the hospital to promote care close to the state of the art, through the adoption of current and evidence-based protocols.	98 (89.9)
Relevant, as involvement in clinical research would make care as a whole updated and evidence-based.	9 (8.3)
Little relevant, as the involvement of my patients in clinical research would take a lot of time, which would make my care in other areas difficult.	0 (0)
Neither relevant nor irrelevant	1 (0.9)
Not relevant at all, as I believe that my knowledge about the main clinical trials in the area is sufficient for an ideal level of care for my patients.	1 (0.9)
For what percentage of patients do you assist in the service, do you offer participation in a clinical trial?	
≤1%	34 (31.2)
2–3%	14 (12.8)
3–5%	19 (17.4)
5–10%	25 (22.9)
>10%	17 (15.6)
What percentage of patients that you assist at the service actually participate in a clinical trial?	
≤1%	52 (47.7)
2–3%	26 (23.8)
3–5%	17 (15.6)
5–10%	9 (8.3)
>10%	5 (4.6)
Total participants	109 (100.0)

**Table 2.** Regression analysis – doesn't work in research field versus work in research field.

Parameters	Level	*N*	Univariate analysis (*n* = 109)			Multivariate analysis (*n* = 109)		
			RR	95% CI	*p*-value	RR	95% CI	*p*-value
Age					0.3586			0.8118
	≤40 years^a^	28						
	40–50 years	42	1.41	0.87–2.27		1.24	0.65–2.36	
	>50 years	13	1.31	0.68–2.52		1.19	0.50–2.82	
Gender					0.9209			0.8541
	Male^a^	46						
	Female	37	1.02	0.66–1.58		1.04	0.67–1.63	
Time since specialisation conclusion					0.3870			0.7155
	≤5 years^a^	10						
	5–10 years	21	1.26	0.59–2.68		1.32	0.59–2.94	
	>10 years	52	1.53	0.78–3.02		1.43	0.59–3.46	
Had contact with clinical research during medical degree					0.6962			0.5173
	Yes^a^	58						
	No	25	0.91	0.57–1.46		0.85	0.51–1.41	
Health service					0.6901			0.6885
	Private^a^	42						
	Public	14	1.31	0.42–2.40		1.31	0.70–2.44	
	Both	27	1.08	0.67–1.76		1.00	0.60–1.69	
Live in the capital					0.6124			0.9879
	Yes^a^	67						
	No	21	1.14	0.69–1.87		1.00	0.57–1.76	

a Reference level; CI: confidence interval; RR: relative risk

**Table 3.** Regression analysis – patience referral (≤5% versus >5%).

Parameters	Level	*N*	Univariate analysis (*n* = 83)			Multivariate analysis (*n* = 83)		
			RR	95% CI	*p*-value	RR	95% CI	*p*-value
Age					0.7189			0.8936
	≤40 years^a^	15						
	40–50 years	21	1.31	0.67–2.55		1.24	0.51–2.99	
	>50 years	6	1.13	0.43–2.90		1.20	0.35–4.08	
Gender					0.8775			0.9908
	Male^a^	24						
	Female	18	0.95	0.51–1.76		1.00	0.52–1.90	
Time since specialisation conclusion					0.8540			0.7587
	≤5 years^a^	6						
	5–10 years	13	1.30	0.49–3.42		0.87	0.29–2.60	
	>10 years	23	1.13	0.46–2.78		0.67	0.20–2.20	
Had contact with clinical research during medical degree					0.6201			0.4127
	Yes^a^	74						
	No	35	1.17	0.62–2.21		1.34	0.66–2.74	
Health service					0.8203			0.9637
	Private^a^	21						
	Public	7	1.31	0.55–3.09		0.97	0.38–2.43	
	Both	14	1.12	0.57–2.21		0.90	0.43–1.88	
Live in the capital					0.2330			0.2317
	Yes^a^	29						
	No	13	1.51	0.78–2.90		1.63	0.73–3.63	
Principal investigator					0.0502			0.3951
	Yes^a^	37						
	No	5	0.43	0.17–1.10		0.64	0.21–1.87	
Linked to a research centre					0.0266			0.1409
	Yes^a^	40						
	No	2	0.27	0.06–1.12		0.34	0.07–1.67	

a Reference level; CI: confidence interval; RR: relative risk

## Supplementary material

### Online survey

This survey is aimed for physicians who work in oncology – screening, diagnosis, treatment, or follow-up of cancer patients. Is this your case?

- Yes
- No

A.1. How old are you? (years old)

A.2. What is your sex?

- Female
- Male

A.3. What is your specialty?

- Oncology
- Radiotherapy
- Oncological surgery
- Other:

A.4. How many years ago did you finish the specialisation?

A.6. In which city do you work?

A.7. During your medical degree (medicine school or medical residency) did you have contact with clinical research?

- Yes
- No

B.1. How relevant do you consider the participation of your patients in clinical research for the quality of care in oncology?

- Not relevant at all, as I believe that my knowledge about the main clinical trials in the area is sufficient for an ideal level of care for my patients.
- Little relevant, as the involvement of my patients in clinical research would take a lot of time, which would make my care in other areas difficult.
- Neither relevant nor irrelevant
- Relevant, as involvement in clinical research would make care as a whole updated and evidence-based.
- Very relevant, as the involvement of the patient in clinical research will help the doctor and the hospital to promote care close to the state of the art, through the adoption of current and evidence-based protocols.

B.2. Does the main oncology service in which you work provide services to the public system? (SUS (Unified Health System) if in Brazil)

- Yes, the service exclusively serves patients from the SUS.
- No, the service exclusively serves patients from the private system.
- Yes, the service serves patients both from the SUS and the private system.

B.3. For what percentage of patients do you assist in the service, do you offer participation in a clinical trial?

- Up to 1%
- 2% to 3%
- 3% to 5%
- Between 5% and 10%
- More than 10%

B.4. What percentage of patients that you assist at the service actually participate in a clinical trial?

- Up to 1%
- 2% to 3%
- 3% to 5%
- Between 5% and 10%
- More than 10%

B.5. Is there a research centre linked to the main oncology service where you work?

- Yes
- No

B.6. Only if the answer is yes to the previous question (B.5) – The research centre linked to the main oncology service in which you work participates in:

- Epidemiological trials only
- Clinical and epidemiological trials
- Clinical trials only

B.7. Are you currently member of a clinical research centre?

- Yes
- No

For participants who answered ‘a.YES’ to question B.6:

B.6.A Data related to the clinical research infrastructure in the region where the researcher works.

B.6.A.1. What is your role at the research centre?

- Principal Investigator
- Sub-investigator
- Physician (I am not an investigator or sub-investigator)

B.6.A.3. Which of the aspects below do you consider the main barrier to conducting clinical research in your region?

- Lack of offer of protocols by CROs/pharmaceutical industry
- Inadequate remuneration for the team (investigator, study coordinator, pharmacist, nurse)
- Lack of incentive from the institution to which the research centre is linked
- Lack of human resources/qualified personnel
- Delay in the approval of protocols by the IEC/IRB to which the centre is linked

B.6.A.4. Which of the aspects below do you consider the main barrier to conducting clinical research in your country?

- Time for the regulatory process (CONEP/ANVISA/CEP system for Brazil)
- The absence of study offers by the pharmaceutical industry
- Lack of interest of hospitals to participate in clinical research protocols
- Lack of knowledge about clinical research on the part of the population leading to difficulty in recruiting research subjects
- Lack of qualification of investigator physicians and multi-professional team

B.6.A.5. What suggestion would you make for your country to participate more in clinical research?

B.6.A.6. Which of the options below best describes your perception of clinical research in your country?

- Little expressive, centralised in the capital cities, restricted to a few physicians, usually opinion leaders, with little participation in clinical trials.
- Quite expressive, restricted to capital cities and opinion leaders, but I believe there are many protocols in which my country participates.
- Few studies in the country, due to a general lack of interest among medical professionals and hospital managers.
- Few protocols in the country, due to the lack of training programs for health care professionals to work with clinical trials.

For participants who answered ‘b.NO’ to question B.6:

B.6.B. Data related to physicians not linked to clinical research

B.6.B.1. Are you interested in working in any area of clinical research?

- Yes
- No

B.6.B.3. Have you ever tried to include the hospital/service where you work in a clinical trial?

- I tried and couldn’t even answer the feasibility (questionnaire applied to the principal investigator and centre about the feasibility to participate in a clinical research)
- I tried, and I managed to answer the feasibility, but the service did not meet any of the feasibility requirements.
- I tried, and the service was qualified in feasibility, but it was not qualified in the visit to the centre.
- I have never tried to include the hospital/service I work in in a clinical trial.

B.6.B.4. What reasons do you consider relevant for not working with clinical research?

- Lack of knowledge on the subject
- Not believing that clinical research brings benefits to cancer patients
- Difficulty engaging in clinical research protocols/procedures
- Unattractive pay standard for this type of work
- Knows the subject well, but doesn’t like this type of work

B.6.B.5. How would you describe, in the current scenario, the oncology service in which you work concerning clinical research?

- There are no professionals qualified in the service
- The hospital is not interested in working with clinical research
- The service is of interest, but there are difficulties such as the shortage of qualified professionals
- The service is of interest, there are some qualified professionals, but it is difficult to be able to participate in the first protocols

B.6.B.6. Which of the options below best describes your perception of clinical research in your country?

- Little expressive, centralised in the capital cities, restricted to a few physicians, usually opinion leaders, with little participation in clinical trials.
- Quite expressive, restricted to capital cities and opinion leaders, but I believe that there are many protocols in which my country participates.
- Few studies in the country, due to a general lack of interest on the part of medical professionals and hospital managers.
- Few protocols in the country, due to the lack of training programs for health care professionals to work with clinical trials.
